# Outcome assessment methods of bioactive and biodegradable materials as pulpotomy agents in primary and permanent teeth: a scoping review

**DOI:** 10.1186/s12903-024-04221-w

**Published:** 2024-04-27

**Authors:** Yasmine Elhamouly, May M. Adham, Karin M L Dowidar, Rania M. El Backly

**Affiliations:** 1 Department of Pediatric and Community Dentistry, Faculty of Dentistry, Pharos University in Alexandria, Canal El Mahmoudia St., Smouha Alexandria, 21648 Egypt; 2 Department of Pediatric Dentistry and Dental Public Health, Faculty of Dentistry, Alexandria University, Champolion St., Azarita, Alexandria, 21527 Egypt; 3 Endodontics, Conservative Dentistry Department and Tissue Engineering Laboratories, Faculty of Dentistry, Alexandria University, Champolion St., Azarita, Alexandria, 21527 Egypt

**Keywords:** Histological outcome, Clinical outcome, Pulpotomy, Bioactive agents, Biodegradable scaffolds, Primary teeth, Permanent teeth

## Abstract

**Background:**

Pulpotomy procedures aiming to preserve and regenerate the dentin-pulp complex have recently increased exponentially due to developments in the field of biomaterials and tissue engineering in primary and permanent teeth. Although the number of studies in this domain has increased, there is still scarcity of evidence in the current literature.

**Objectives:**

(1) Report the methods of outcome assessment of pulpotomy clinical trials in both primary and permanent teeth; (2) Identify the various bioactive agents and biodegradable scaffolds used in pulpotomy clinical trials in both primary and permanent teeth.

**Materials and methods:**

A scoping review of the literature was performed, including a search of primary studies on PubMed, Scopus, Web of Science, ProQuest and Clinicaltrials.gov. A search for controlled trials or randomized controlled trials published between 2012 and 2023 involving primary or permanent teeth receiving partial or full pulpotomy procedures using bioactive/regenerative capping materials was performed.

**Results:**

127 studies out of 1038 articles fulfilled all the inclusion criteria and were included in the current scoping review. More than 90% of the studies assessed clinical and radiographic outcomes. Histological, microbiological, or inflammatory outcomes were measured in only 9.4% of all included studies. Majority of the studies (67.7%) involved primary teeth. 119 studies used non-degradable bioactive cements, while biodegradable scaffolds were used by 32 studies, natural derivates and plant extracts studies were used in only 7 studies. Between 2012 (4 studies) and 2023 (11 studies), there was a general increase in the number of articles published. India, Egypt, Turkey, and Iran were found to have the highest total number of articles published (28, 28,16 and 10 respectively).

**Conclusions:**

Pulpotomy studies in both primary and permanent teeth relied mainly on subjective clinical and radiographic outcome assessment methods and seldom analyzed pulpal inflammatory status objectively. The use of biodegradable scaffolds for pulpotomy treatments has been increasing with an apparent global distribution of most of these studies in low- to middle-income countries. However, the development of a set of predictable outcome measures as well as long-term evidence from well conducted clinical trials for novel pulpotomy dressing materials are still required.

**Supplementary Information:**

The online version contains supplementary material available at 10.1186/s12903-024-04221-w.

## Background

Pulpotomy is a minimally invasive vital pulp therapy in which a portion of an infected vital pulp is amputated or removed to preserve the vitality and function of the residual pulp tissue [1]. It is currently considered a common practice for asymptomatic, cariously exposed pulps of primary and young permanent teeth. This helps maintain the integrity of primary teeth that have inflammation limited to the coronal pulp, preserve the vitality of the radicular pulp, and ultimately retain the tooth until its normal exfoliation [2]. On the other hand, long-term preservation of permanent teeth requires a tooth with a favorable crown-to-root ratio and dentin walls thick enough to withstand normal function. Therefore, pulp preservation in immature permanent teeth with partial or full pulpotomy is also a paramount goal, since conventional root canal treatment inhibits the development of physiological dentin, exposing the thin canal walls to fracture of the root [2].

The clinical relevance of the inflammation-regeneration interplay has been further emphasized by the successful clinical and radiographic outcomes of pulpotomized mature teeth diagnosed with irreversible pulpitis in numerous studies. A treatment modality that was once considered an interim or emergency treatment at best is now being suggested as an alternative treatment modality to root canal treatment [3, 4]. Indeed, this has led to a plethora of clinical studies employing vital pulp therapy procedures as viable permanent therapeutic options for the mature permanent tooth with an inflamed dental pulp. In particular, pulpotomy procedures have been recently advocated as a viable treatment modality for mature permanent teeth diagnosed with irreversible pulpitis as yet another pillar of minimally invasive endodontics. While pulpotomy has been long utilized in pediatric dentistry for non-symptomatic primary teeth and in immature permanent teeth to preserve the radicular pulp and allow for apexogenesis to continue, this concept is relatively new for mature teeth. Several systematic reviews have indeed demonstrated significant success rates in pulpotomies of mature permanent teeth [3, 5, 6].

The outcome of primary teeth pulpotomy is commonly assessed clinically by the absence of pain, swelling, and sinus tract, or by clinical tests such as palpation, percussion, and mobility. Also assessed radiographically by the presence of normal periodontal ligament (PDL), absence of furcation or apical radiolucency, or evidence of internal/external resorption [7]. In addition to the forementioned criteria, the rationale in young permanent teeth undergoing partial or complete pulpotomy is that the remaining vital pulp enables the continuation of normal root development and apexogenesis, as determined by periodic radiographic evaluation [8].

Notwithstanding these recent revelations, current diagnostic terminology of pulpal status has been challenged as has the search for a consensus on how outcome assessment is interpreted following vital pulp therapy procedures in mature permanent teeth with inflamed pulps [9]. This in turn has triggered the search for more predictable assessors of pulpal inflammatory status, aiming to provide more predictable treatment outcomes. Another challenging area in endodontics is a lack of a universal core outcome set [10]. This is further complicated by the heterogeneity of reported outcomes and the lack of standardization particularly in the scope of vital pulp therapy modalities. Patient-reported outcomes are generally centred only around pain, ignoring other parameters such as tooth survival and oral health-related quality of life (OHRQoL). These outcomes, in addition to clinician-reported ones, should provide the basis for developing a set of core outcomes with consensus among clinicians [11].

One of the areas that has served to propel forward this new direction is the immense evolution of hydraulic cements, or calcium silicate cements, by providing bioactive, antimicrobial, and biocompatible dressings for the inflamed pulp in addition to providing an immediate seal [12]. The superior outcomes reported from clinical trials that used hydraulic calcium silicate cements are undeniable; however, well-controlled, long-term, high quality randomized clinical trials are still needed to make definitive selections on the best material to use [11]. In spite of the presence of numerous advanced hydraulic cements currently available in the market, none of these is capable of being completely replaced with new pulp tissue following a coronal pulpotomy, i.e. engineering a new functional dentin-pulp complex will remain the holy grail for any tissue regeneration strategy. Stemming from that, biomimetic biodegradable scaffolds whether in a cell-based or cell-free approach, have also received much attention as therapeutic agents following pulpotomy procedures [4]. 

Similarly, pulpotomy agents in primary teeth have evolved over the last century from the action of devitalization to preservation of the radicular pulp and ultimately to tissue regeneration. A variety of regenerative agents, including bioactive cements, biodegradable scaffolds, and natural derivatives, have also been used for regenerating the dentin-pulp complex in pulpotomized primary teeth [13]. The American Academy of Pediatric Dentistry’s clinical guidelines has recommended mineral trioxide aggregate (MTA) as the medicament of choice for teeth expected to be retained for 24 months or more. Other tricalcium silicates have conditional recommendations, and they recommended against the use of calcium hydroxide for pulpotomy of primary teeth [2]. However, the success of pulpotomy procedures depends on many factors other than the biological effect of the pulpotomy agent; these include the diagnosis of the preoperative and intraoperative pulp status, caries topography and extension, technique, final restoration, and the operator’s experience [14].

Although there has been a steep increase in the number of studies utilizing bioactive cements and tissue engineering approaches for dentin/pulp regeneration in primary and permanent teeth, there are still numerous gaps of knowledge, and the overall quality of evidence is low [11]. These gaps include the lack of objective tools for assessment of the true inflammatory status of the pulp as well as the absence of a clear core outcome set of measures for analyzing the results for both primary and permanent teeth. Moreover, the contribution of tissue engineering scaffolds to pulpotomy clinical trials is unclear for both dentitions. Hence, this scoping review aimed to map the existing clinical evidence on the outcome of pulpotomy procedures in both primary and permanent teeth using bioactive cements and biodegradable scaffolds. The objectives were to: (1) Report the methods of outcome assessment of pulpotomy clinical trials in both primary and permanent teeth; (2) Identify the different bioactive agents and biodegradable scaffolds employed in pulpotomy clinical trials in both primary and permanent teeth.

## Materials and methods

This scoping review was carried out following the Joanna Briggs Institute (JBI) Methodology for Scoping Reviews [15]. The focused PCC question was: What is the available evidence on the outcome assessment methods of pulpotomy in primary and permanent teeth using bioactive agents and biodegradable scaffolds?

### Literature search and study selection

An electronic literature search was conducted using MEDLINE (via PubMed), Web of Science, Scopus, ProQuest, and clinicaltrials.gov between the inception date and October 2023. The search strategy adopted for each database is presented in Table 1.

**Table 1:** Search strategy for databases included in the review

Database	Search terms	Initial number of results
PubMed	((((((((Bioactive) OR (regenerative) OR (Pulp Capp*)) OR (pulp dress*)) OR (calcium silicate)) OR (non-degradable bioactive materials)) OR (degradable natural and synthetic tissue engineering scaffolds)) AND (pulpotomy)) AND ((“Tooth, Deciduous“[Mesh])) OR (“Dentition, Permanent“[Mesh]) AND (pulp) AND (“Histology“[MeSH Terms] OR “Clinical Trial“[Publication Type])) (y_10[Filter])	40
Scopus	( ( *bioactive* ) OR ( *regenerative* ) OR ( *calcium* AND *silicate* ) ) AND ( *pulpotomy* ) AND ( ( *primary* ) OR ( *permanent* ) AND ( *teeth* ) ) AND ( *clinical* ) AND ( LIMIT-TO ( SRCTYPE , *“j"* ) ) AND ( LIMIT-TO ( DOCTYPE , *“ar"* ) ) AND ( LIMIT-TO ( SUBJAREA , *“DENT"* ) ) AND ( LIMIT-TO ( PUBYEAR , *2023* ) OR LIMIT-TO ( PUBYEAR , *2022* ) OR LIMIT-TO ( PUBYEAR , *2021* ) OR LIMIT-TO ( PUBYEAR , *2020* ) OR LIMIT-TO ( PUBYEAR , *2019* ) OR LIMIT-TO ( PUBYEAR , *2018* ) OR LIMIT-TO ( PUBYEAR , *2017* ) OR LIMIT-TO ( PUBYEAR , *2016* ) OR LIMIT-TO ( PUBYEAR , *2015* ) OR LIMIT-TO ( PUBYEAR , *2014* ) OR LIMIT-TO ( PUBYEAR , *2013* ) OR LIMIT-TO ( PUBYEAR , *2012* ) ) AND ( LIMIT-TO ( LANGUAGE , *“English"* ) ) AND ( LIMIT-TO ( EXACTKEYWORD , *“Human"* ) OR LIMIT-TO ( EXACTKEYWORD , *“Humans"* ) )	372
Web of science	(((((((((((ALL=(Bioactive)) OR ALL=(regenerative)) OR ALL=(pulp capping)) OR ALL=(pulp dressing)) OR ALL=(calcium silicate)) OR ALL=(non-degradable bioactive materials))) OR ALL=(degradable natural and synthetic tissue engineering scaffolds))) AND ALL=(pulpotomy)) AND ALL=((Primary OR Permanent) AND (teeth))) AND ALL=((Histology OR Clinical)) Filters: from 2012–2023	171
ProQuest	(Bioactive OR regenerative) AND (pulpotomy) AND (primary OR permanent teeth) AND (clinical OR histological) Applied filters: Last 10 years	365
Clinicaltrials.gov	Pulpotomy (as the condition) Applied filters: Recruiting, Not yet recruiting, Active, not recruiting, Completed, Terminated Studies | Interventional Studies | pulpotomy | Start date from 01/01/2012 to 10/31/2023	90

After exporting the search results from the databases to Zotero, duplicates were removed. Next, a title-and-abstract and a full-text screening phase were performed by two reviewers in an independent and duplicated manner to identify potentially eligible studies. References of all included articles were also screened to avoid any missing eligible studies. Records retrieved from ProQuest and Clinicaltrials.gov with published results in peer-reviewed journals were considered as duplicates. Regarding the date of the status of studies retrieved from Clinicaltrials.gov, the “actual completion date” was considered for completed studies while the “last update posted” was considered for studies that had either an unknown status, active, or recruiting. Agreement between reviewers in the selection process was calculated by the Cohen’s Kappa statistics (k = 0.8). Any discrepancies were resolved by a third reviewer.

### Eligibility criteria

#### Inclusion criteria

- Time: 2012–2023.
- Age: no filter.
- Study type: primary research (Controlled trials, randomized controlled trials).
- Studies executing partial/full pulpotomy procedures.
- Studies done on primary and/or permanent teeth.
- Studies including bioactive/regenerative capping materials.

#### Exclusion criteria

- No abstract available.
- Not in English language.
- Published prior to 2012.
- Vital pulp therapy modalities including indirect/direct pulp capping.
- Studies conducted on non-vital teeth.
- Studies comparing pulpotomy with other vital pulp therapy procedures.
- Studies assessing success and failure outcomes of pulpotomy that are not dependent on the type of pulp dressing material.
- Case reports and case series.
- Secondary research; reviews whether systematic or otherwise and surveys.
- Position statements and clinical guidelines.
- Papers that cannot be fully accessed.
- Single arm studies.

#### Data collection and analysis

Two independent reviewers extracted data from the included studies into a standardized data extraction table, which was then subsequently counter-checked by another two reviewers. Data extracted for each paper included: study reference (author(s), year of publication, title, name of journal, and country where the study was conducted), study design, follow-up period, initial diagnosis representing pulp exposure type, arms of the study, outcome measures assessed, type of material used, sample size, and whether the study involved primary or permanent teeth. Details of the studies included are presented in Table 2.

**Table 2:** Studies included in the review *(arranged alphabetically according to author name)*

Author, year	Country	Study design	Sample size	Follow-up duration	Outcome measures	Materials used	Type of exposure	Primary/ Permanent	Pre-operative Pulp Status
Abdelwahab D, 2023 [54]	Egypt	RCT	60	1,3,6,12 months	Clinical and radiographic	Totalfill® BC RRM™ Fast Set Putty and MTA WHITE	Carious	Primary	Reversible pulpitis
Abd Al Gawad R and Hanafy R. 2021 [55]	Egypt	RCT	72	3,6,12 months	Clinical and radiographic	NHA (Straumann Bone Ceramic), MTA, Formocresol	Carious OR traumatic	Primary	Reversible pulpitis
Abdel Maksoud E, 2023 [56]	Egypt	RCT	36	3,6,9,12 months	Clinical, radiographic, microbiological	Hyaluronic Acid, Amniotic Membrane Allograft, Mineral Trioxide Aggregate	Carious	Primary	Reversible pulpitis
Aboul Kheir M et al., 2020 [57]	Egypt	RCT	30	12 months	Clinical and radiographic	Chitosan scaffold, MTA	Carious	Permanent	Irreversible pulpitis
Abuelniel G et al., 2020 [58]	Egypt	RCT	50	18 months	Clinical and radiographic	MTA, Biodentine	Traumatic	Immature anterior permanent teeth	Reversible pulpitis
Abuelniel G et al., 2021 [59]	Egypt	RCT	60	6, 12 and 18 months	Clinical and radiographic	MTA, Biodentine	Carious	Immature permanent teeth	Reversible pulpitis
Airen P et al., 2012 [60]	India	NRS	70	24 months	Clinical and radiographic	MTA, Formocresol	Carious	Primary	Reversible pulpitis
Airsang A et al., 2022 [61]	India	RCT	60	6 months and 1 year	Clinical and radiographic	NeoMTA, Biodentine	Carious	Mature permanent	Irreversible pulpitis
Akcay M et al., 2014 [62]	Turkey	RCT	128	12 months	Clinical and radiographic	Calcium hydroxide, MTA	Carious	Primary	Reversible pulpitis
Aksoy B et al., 2022 [63]	Turkey	RCT	105	2 years (6,12,18 and 24)	Clinical and radiographic	Zinc oxide–eugenol, Calcium hydroxide, MTA	Carious	Primary	Reversible pulpitis
Alacam A, 2017 [64]	Turkey	RCT	54	12 months	Clinical and radiographic	Biodentine, Calcium hydroxide, MTA	Carious	Young permanent molars	Reversible pulpitis
Alajaji N, 2021 [65]	Iran	NRS	469	4 years	Clinical and radiographic	MTA, Ferric sulfate, Biodentine	Carious	Primary	Reversible pulpitis
Alamoudi N et al., 2018 [66]	KSA	RCT	106	3, 6 and 12 months	Clinical and radiographic	Low-level laser, Formocresol	Carious	Primary	Reversible pulpitis
Alamoudi N, 2016 [67]	KSA	RCT	112	6 and 12 months	Clinical and radiographic	Biodentine, Formocresol	Carious	Primary	Reversible pulpitis
Aljabban et al., 2021 [133]	Syria	RCT	24	8 weeks	Clinical and histological	MTA, PRF	Sound premolar teeth scheduled for orthodontic extraction	Permanent	Normal pulp
Alnassar I et al., 2023 [68]	Syria	RCT	40	1 week, 3 months, 6 months, 9 months, and 1 year	Clinical and radiographic	MTA, Bioceramic putty	Carious	Primary	Reversible pulpitis
Alzoubi H et al., 2021 [69]	Syria	NRS	35	3, 6, 12 months histological evaluation after 3 months	Clinical and radiographic	Portland cement, MTA	Carious	Primary	Reversible pulpitis
Anandan V et al., 2021 [70]	India	NRS	30	2,4 and 6 months	Clinical and radiographic	Formocresol BioFil-AB Collagen Particles	Carious	Primary	Reversible pulpitis
Aripirala M et al., 2021 [71]	India	RCT	100	12 months	Clinical and radiographic	Simvastatin gel, 940 nm diode laser	Carious	Primary	Reversible pulpitis
Asgary S et al., 2012 [72]	Iran	RCT	413	12 months	Clinical and radiographic	MTA, calcium enriched cement (CEM)	Carious	Permanent	Irreversible pulpitis
Asgary S et al., 2022 [73]	Iran	RCT.	154	2 years and pain was assessed upto one week	Clinical and radiographic	Proroot MTA, CEM	Carious	Permanent	Reversible pulpitis OR Irreversible pulpitis
Awad S, 2021 [74]	Egypt	RCT	17	2 years	Clinical and radiographic	Biodentine, Calcium Hydroxide, PRF	Carious	Infected immature permanent molars	NOT MENTIONED
Awawdeh L et al., 2018 [75]	Jordan	RCT	68	3 years	Clinical and radiographic	Biodentine, MTA	Carious	Permanent	Reversible pulpitis
Bakhtiar H et al., 2017 [152]	Iran	RCT	27	8 weeks	Clinical, radiographic, histological	Theracal, Biodentine, proroot MTA	Traumatic	Permanent third molars	Normal pulp
Bakhtiar H et al., 2018 [153]	Iran	RCT	22	1 and 8 weeks	Clinical and histological	Retro-MTA, pro-root MTA	Sound teeth scheduled for extraction	Permanent	Normal pulp
Bani M et al., 2022 [76]	Turkey	RCT	62	24 months	Clinical and radiographic	MTA, Biodentine	Carious	Primary molars	Reversible pulpitis
Bayoumi N, 2022 [77]	Egypt	RCT	40	12 months	Clinical and radiographic	Sterile medicated collagen particles, Biofil-AB, Biodentine	Carious	Primary	Reversible pulpitis
Bhagat D et al., 2017 [154]	India	RCT	30	6 months	Histological	MTA, Portland cement	Traumatic	Premolars scheduled for extraction	Normal pulp
Brar K et al., 2020 [78]	USA	NRS	102	3 years	Clinical and radiographic	Ferric sulfate, Biodentine	Carious	Primary	Reversible pulpitis
Carti O and Oznurhan F, 2017 [79]	Sivas, Turkey	RCT	50	12 months	Clinical and radiographic	Biodentine, MTA	Carious	Primary	Reversible pulpitis
Caruso S et al., 2018 [80]	Italy	NRS	400	9 and 12 months	Clinical and radiographic	Biodentine, Calcium hydroxide	Carious	Primary	Reversible pulpitis
Celik B et al., 2013 [81]	Turkey	RCT	139	24 months	Clinical and radiographic	MTA, Calcium hydroxide	Carious	Primary	Reversible pulpitis
Celik B et al., 2019 [82]	Turkey	RCT	44	3,6,12, 18 and 24 months	Clinical and radiographic	MTA, Biodentine	Carious	Primary	Reversible pulpitis
Chailertvanitkul P et al., 2014 [83]	Thailand	RCT	84	24 months	Clinical and radiographic	MTA, Calcium hydroxide	Carious	Permanent	Reversible pulpitis
Chak R et al., 2022 [84]	India	RCT	60	3, 6, 9, and 12 months	Clinical and radiographic	3Mixtatin, MTA	Carious	Primary	Reversible pulpitis
Chen J, 2017 [85]	USA	RCT	56	6, 9 and 12 months	Clinical and radiographic	MTA, Ferric Sulfate	Carious	Primary molars	Reversible pulpitis
Clancy M, 2018 [86]	USA	RCT	60	2 years with follow-up every 6 months	Clinical and radiographic	Biodentine, Formocresol	Carious	Primary	Reversible pulpitis
Cogulu D, 2019 [87]	Turkey	RCT	57	6, 12,18 months	Expression levels of MMP-2, 8 and 9; and clinical and radiographic	MTA, Biodentine	Carious	Primary	Reversible pulpitis
Cordell S, 2019 [88]	USA	RCT	50	6 and 12 months	Clinical and radiographic	NeoMTA, 15.5% ferric sulfate solution	Carious	Primary	Reversible pulpitis
de Lima S et al., 2020 [89]	Brazil	RCT	70	seven days, and at 1, 3, 6 and 12 months	Clinical and radiographic	Bio-C Pulpo, MTA	Carious	Primary	Reversible pulpitis
Eid A et al., 2022 [90]	Syria	RCT	63	12 months	Clinical and radiographic	MTA (MM-MTA), nano-hydroxyapatite, platelet-rich fibrin	Carious	Young permanent molars	Reversible pulpitis
El Meligy O et al., 2016 [155]	KSA	RCT	112	6 months	Clinical and radiographic	Biodentine, Formocresol	Carious	Primary	Reversible pulpitis
El Meligy O et al., 2019 [91]	Saudi Arabia	RCT	112	3,6,12 months	Clinical and radiographic	Biodentine, Formocresol	Carious	Primary	Reversible pulpitis
Elbardissy A, 2018 [92]	Egypt	RCT	43	3,6,9,12 months	Clinical and radiographic	Biodentine, Formocresol	Carious	Primary	Reversible pulpitis
El-desouky S, 2023 [93]	Egypt	RCT	30	6,12 months	Clinical and radiographic	Eggshell Powder freshly mixed with Tea Tree Oil, Biodentine, MTA	Carious	Primary	Reversible pulpitis
Elhamouly Y et al., 2021 [18]	Egypt	Interim analysis /terminated RCT	19	12 months	Clinical, radiographic, histological	Biodentine, bioactive glass	Carious	Primary	Reversible pulpitis
Elheeny A, 2023 [94]	Egypt	RCT	128	12, 18 months	Clinical and radiographic	Simvastatin, MTA	Carious	Immature permanent teeth	Reversible pulpitis
Elsayed S, 2023 [136]	Egypt	RCT	40	3,6 months	Clinical and radiographic	Biofil-AB, Biodentine	Carious	Primary	Reversible pulpitis
Elshaer N, 2021 [95]	Egypt	RCT	40	12 months	Clinical and radiographic	Protooth MTA, MTA	Carious	Primary	Reversible pulpitis
Elshamy SH, 2022 [96]	Egypt	RCT	38	12 months	Clinical and radiographic	Calcium hydroxide, Biodentine	Carious	Young permanent molars	Reversible pulpitis
Eltantawy W, 2023 [97]	Egypt	RCT	96	18 months	Clinical and radiographic	Biodentine, hyaluronic acid, Formocresol	Carious	Primary	Reversible pulpitis
Eshghi A et al., 2022 [98]	Iran	RCT	52	3,6, 9, 12 months	Clinical and radiographic	MTA, Biodentine	Carious	Primary	Reversible pulpitis
Fernandez C et al., 2013 [99]	Spain	RCT	100	24 months	Clinical and radiographic	Formocresol, MTA, ferric sulphate, and NaOCl	Carious	Primary	Reversible pulpitis
Fouad W et al., 2019 [100]	Egypt	NRS	84	12 months	Clinical and radiographic	Biodentine, MTA	Carious	Primary	Reversible pulpitis
Frenkel G et al., 2012 [101]	Israel	NRS	86	47 months	Clinical and radiographic	Ferric Sulphate, MTA	Carious	Primary	Reversible pulpitis
Gaber R et al., 2022 [156]	Egypt	RCT	20	6 months	Clinical and radiographic	MTA, Theracal	Carious	Primary	Reversible pulpitis
Gamal D, 2023 [103]	Egypt	RCT	24	1 year	Clinical and radiographic	Wellroot PT and MTA	Carious	Primary	Reversible pulpitis
Ghent University, 2020 [104]	Belgium	RCT	36	12 months	Clinical and radiographic	Totalfill Bioceramic Root Repair Material®, MTA	Carious	Primary teeth	Reversible pulpitis
Grewal N et al., 2016 [105]	India	RCT	40	12 months	Clinical and radiographic	Biodentine, Calcium hydroxide	Carious	Primary molars	Reversible pulpitis
Guven Y et al., 2016 [106]	Turkey	RCT	116	24 months	Clinical and radiographic	Proroot MTA, MTA-Plus, Biodentine, ferric sulfate	Carious	Primary molars	Reversible pulpitis
Hadassah Medical Organization, 2021 [107]	Israel	NRS	60	36 months	Clinical and radiographic	MedCem MTA, Formocresol	Carious	Primary	Reversible pulpitis
Haideri S et al., 2021 [108]	India	NRS	80	3,6,12 months	Clinical and radiographic	Formocresol, Mineral Trioxide Aggregate, Electrocautery, Bioactive Glass	Carious	Primary	Reversible pulpitis
Hugar S et al., 2017 [109]	India	RCT	60	60 months	Clinical, radiographic, histological	MTA, Formocresol	Carious	Primary	Reversible pulpitis
Ildes G et al., 2022 [110]	Turkey	RCT	130	1,3,6 and 12 months	Clinical and radiographic	0.5% Hyaluronic Acid gel, Formocresol, 20% Ferric sulphate	Carious	Primary molars	Reversible pulpitis
Jayam C et al., 2018 [111]	India	RCT	100	24 months	Clinical and radiographic	MTA, Formocresol	Carious	Primary	Reversible pulpitis
Jimeno F et al., 2022 [112]	Spain	RCT	108	12 months	Clinical and radiographic	MTA ProRoot, MTA HP Repair, Biodentine	Carious	Primary	Reversible pulpitis
Joo Y et al., 2023 [113]	Korea	RCT	153	3, 6, and 12 months	Clinical and radiographic	MTA, Wellroot PT, Proroot MTA	Carious	Primary	Reversible pulpitis
Juneja P and Kulkarni S, 2017 [114]	India	RCT	51	18 months	Clinical and radiographic	MTA, Biodentine, Formocresol	Carious	Primary molars	Reversible pulpitis
Junqueira M et al., 2017 [115]	Brazil	NRS	31	18 months	Clinical and radiographic	MTA and 15.5% Ferric Sulfate	Carious	Primary molars	Reversible pulpitis
Kakarla P et al., 2013 [102]	India	RCT	40	24 months	Histological	Pulpotec, Biofil-AB	Retained sound and indicated for orthodontic extraction	Primary	Reversible pulpitis
Kalra M et al., 2017 [116]	India	RCT	60	12 months	Clinical and radiographic	Fresh Aloe vera barbadensis plant extract, MTA	Carious	Primary molars	Reversible pulpitis
Kang C et al., 2015 [117]	Korea	RCT	151	12 months	Clinical and radiographic	Proroot MTA, OrthoMTA, RetroMTA	Carious	Primary molars	Reversible pulpitis
Kang C et al., 2017 [118]	Korea	RCT	104	1,3,6, and 12 months	Clinical and Radiographic	Proroot MTA, OrthoMTA, RetroMTA	Carious OR traumatic	Permanent	Reversible pulpitis
Kang C et al., 2021 [119]	Korea	RCT	104	1, 3, 6, and 12 months and at 48–78 months	Clinical and radiographic	ProRoot MTA, OrthoMTA, RetroMTA	Carious	Mature permanent teeth	Reversible pulpitis
Kathal S et al., 2017 [120]	India	RCT	40	12 months	Clinical and radiographic	MTA, antioxidant mix	Carious	Primary molars	Reversible pulpitis
Keles S, 2018 [121]	Turkey	RCT	96	3,6, 9 and 18 months	Clinical and radiographic	OrthoMTA, RetroMTA and Ferric sulfate	Carious	Primary	Reversible pulpitis
Keswani D et al., 2014 [122]	India	RCT	70	24 months	Clinical and radiographic	MTA, PRF	Carious	Immature Permanent	Reversible pulpitis
Koruyucu M, 2016 [123]	Turkey	RCT	200	3 years	Clinical and radiographic	ProRoot MTA, Biodentine	Carious	Primary	Reversible pulpitis
Kumar V et al., 2016 [124]	India	RCT	54	12 months	Clinical and radiographic	Calcium hydroxide, MTA, platelet-rich fibrin	Carious	Permanent molars	Irreversible pulpitis
Lourenco N et al., 2016 [157]	Brazil	RCT	25	3 months	Histological and immunohistochemistry	Formocresol, Calcium hydroxide, MTA, Portland cement	Carious	Primary	Reversible pulpitis
Madan K et al., 2020 [125]	India	NRS	40	3,6,12 months	Clinical and radiographic	MTA, propolis	Carious	Primary	Reversible pulpitis
Magdy M, 2020 [126]	Egypt	RCT	36	18 months	Clinical and radiographic	MTA, Biodentine	Carious	Primary	Reversible pulpitis
Mahmoud S, 2022 [127]	Egypt	RCT	130	12 months	Clinical and radiographic	MTA, Formocresol	Carious	Primary teeth	Reversible pulpitis
Manhas M et al., 2019 [135]	India	RCT	30	1,3,6, months	Clinical and radiographic	MTA, Calcium hydroxide, PRF	Carious	Primary	Reversible pulpitis
Manohar S et al., 2022 [21]	India	NRS	120	6, 12, 18, and 24 months	Clinical and radiographic	Biodentine, MTA Plus, Retro MTA, CEM cement	Carious	Primary	Reversible pulpitis
Mehrvarzfar P et al., 2017 [134]	Iran	RCT with histologic assessment	39	6 weeks	Clinical, radiographic, histological	MTA, Treated dentin matrix scaffold	Traumatic	Permanent third molars	Normal pulp
Mentes A, 2020 [22]	Turkey	RCT	120	1,3,6,12 months	Clinical and radiographic	Formocresol, ferric sulphate, and 0.5% hyaluronic acid	Carious	Primary	Reversible pulpitis
Nageh M, 2021 [23]	Egypt	RCT	120	24 h, 48 h, 1 week, every 3 months for 12 months	Clinical and radiographic	Biodentine, PRF, MTA, Portland cement	Carious	Permanent	Irreversible pulpitis
Nagy P, 2017 [24]	Egypt	RCT	22	12 months	Clinical and radiographic	MTA, theracal	Carious	Permanent	NOT MENTIONED
Najmi N, 2022 [25]	Pakistan	RCT	114	12 months	Clinical and radiographic	PRF, MTA, calcium hydroxide	Carious	Mature permanent	Irreversible pulpitis
Neto J, 2017 [167]	Brazil	RCT	30	6 months	Clinical and radiographic	Formocresol, PBS CIMMO cement, Zinc oxide	Carious	Primary	Reversible pulpitis
Nguyen T et al.,2014 [26]	Canada	RCT	48	40 months	Clinical and radiographic	MTA, Ferric sulfate	Carious	Primary	Reversible pulpitis
Nosrat A et al., 2012 [27]	Iran	RCT	51	12 months	Clinical and radiographic	MTA, CEM cement	Carious	Permanent	Irreversible pulpitis
Oliveira T et al., 2013 [28]	Brazil	RCT	45	6,12 and 24 months	Clinical, radiographic, histological	MTA, Calcium hydroxide, Portland cement	Carious	Primary	Reversible pulpitis
Özgür B, et al. 2017 [29]	Turkey	RCT	80	6, 12, 18, and 24 months.	Clinical and radiographic	MTA, calcium hydroxide	Carious	Immature permanent	Reversible pulpitis
Patidar S et al., 2017 [138]	India	RCT	50	6 months	Clinical and radiographic	PRF, MTA	Carious	Primary molars	Reversible pulpitis
Perea M et al., 2017 [30]	Spain	RCT	212	48 months	Clinical and radiographic	Formocresol, MTA	Carious	Primary molars	Reversible pulpitis
Petel R et al., 2021 [31]	Israel	RCT	136	24–48 months	Clinical and radiographic	Formocresol, Portland cement	Carious	Primary	Reversible pulpitis
Prasad M et al., 2017 [139]	India	NRS	30	9 months	Clinical and radiographic	Amniotic, Formocresol	Carious	Primary	Reversible pulpitis
Pratima B et al., 2018 [32]	India	NRS	40	6, 12 months	Clinical and radiographic	Diode laser, MTA	Carious	Primary	Reversible pulpitis
Rajasekharan S et al., 2017 [33]	Belgium	RCT	82	18 months	Clinical and radiographic	Biodentine, proroot MTA	Carious	Primary	Reversible pulpitis
Rao Q et al., 2020 [34]	China	NRS	205	6–8 weeks, 1 year then yearly for 5 years	Clinical and radiographic	iRoot BP Plus, calcium hydroxide	Traumatic	Mature permanent teeth	Reversible pulpitis
Rojaramya K et al., 2022 [35]	India	RCT	60	2 years	Clinical and radiographic	MTA, propolis	Carious	Primary	Reversible pulpitis
Rubanenko M, et al. 2019 [36]	Israel	RCT	72	48 months	Clinical and radiographic	biodentine, Formocresol	Carious	Primary	Reversible pulpitis
Sajadi F et al., 2021 [168]	Iran	RCT	38	3,6, months for clinical and radiographic. pain was evaluated up to 10 days after treatment	Clinical and radiographic	Ferric Sulfate, Calcium-Enriched Mixture Cement (CEM)	Carious	Primary	Reversible pulpitis
Sharaan M and Ali A, 2022 [37]	Egypt	RCT	40	7 days and 3, 6 and 12 months	Clinical and radiographic	MTA, CEM	Carious	Permanent	Irreversible pulpitis
Sharaf R et al., 2021 [150]	Egypt	RCT	90	6 months	Clinical and radiographic	Turmeric extract, Thymus Vulgaris extract, Nigella Sativa extract, aloe vera extract, Formocresol	Carious	Primary	Reversible pulpitis
Sherif R, 2019 [131]	Egypt	RCT	38	12 months	Clinical and radiographic	PRF, Biodentine, diode laser	Carious	Permanent Molars	Irreversible pulpitis
Silva L et al., 2019 [151]	Brazil	RCT	45	3,6 and 12 months	Clinical and radiographic	MTA, Calcium hydroxide, polyethylene glycol	Carious	Primary	Reversible pulpitis
Singh R et al., 2020 [38]	India	NRS	60	12 months	Clinical and radiographic	Calcium hydroxide, MTA, PRF	Carious	Permanent	Irreversible pulpitis
Singh, D et al., 2023 [39]	India	RCT	64	1, 3, 6, and 12 months	Clinical and radiographic	MTA, premixed bioceramic putty	Carious	Mature permanent	Reversible pulpitis
Suez Canal University, 2022 [40]	Egypt	RCT	60	12 months	Clinical and radiographic	Biodentine, Simvastatin	Carious	Primary	Reversible pulpitis
Surinder et al.,2021 [132]	India	RCT	60	9 months	Clinical and radiographic	MTA, Biodentine, Platelet Rich Fibrin	Carious	Permanent	Irreversible pulpitis
Taha N et al., 2022 [41]	Jordan	RCT	164	6 and 12 months	Clinical and radiographic	Proroot MTA, Biodentine, totalfill	Carious	Permanent	Reversible pulpitis OR Irreversible pulpitis
Taha N, 2017 [42]	Jordan	RCT	150	6 m, 1 year then yearly for 5 years	Clinical and radiographic	MTA, Biodentine, non-specified Bioceramic	Carious	Permanent	Reversible pulpitis OR Irreversible pulpitis
Togaru H et al., 2016 [43]	India	RCT	90	12 months	Clinical and radiographic	Biodentine, MTA	Carious	Primary molars	Reversible pulpitis
Tozar K and Almaz M, 2019 [44]	Turkey	RCT	90	12 months	Clinical and radiographic	Laser, MTA	Carious	Immature permanent	Reversible pulpitis
Tzanetakis G et al., 2023 [45]	Greece	RCT	137	7days-2 years	Clinical and radiographic	MTA, Total Fill BC	Carious	Mature permanent	Irreversible pulpitis
Uesrichai N et al., 2019 [46]	Thailand	Non-inferiority RCT	69	every 6 m for mean follow up of 32.2 +/-17.9 months	Clinical and radiographic	Biodentine, Proroot MTA	Carious	Permanent	Irreversible pulpitis
Université de Montréal, 2016 [47]	Canada	RCT	180	12 months	Clinical and radiographic	Biodentine, Formocresol	Carious	Primary	Reversible pulpitis
Vafaeia A et al., 2022 [48]	Iran	NRS	316	28.2 ± 2.7 months	Clinical and radiographic	Protooth calcium silicate cement, MTA	Carious	Immature permanent teeth	Reversible pulpitis
Venugopal N et al., 2019 [137]	India	RCT	90	6 months	Clinical, radiographic, histological	Formocresol, propolis, Platelet derived growth factor (PDGF)/scaffold	Carious	Primary molars	Reversible pulpitis
Vilella-Pastor S et al., 2021 [49]	Spain	RCT	84	6,12,18,24 months	Clinical and Radiographic	MTA, Biodentine	Carious OR traumatic	Primary	Reversible pulpitis
Vu T et al., 2020 [50]	Vietnam	NRS	50	12 months	Clinical and radiographic	Acemannan, MTA	Carious OR traumatic	Permanent	Reversible pulpitis
Wassel M, 2019 [51]	Egypt	RCT	60	12 months	Clinical and radiographic	Theracal, Formocresol	Carious	Primary	Reversible pulpitis
Yang Y et al., 2020 [52]	China	RCT	110	1, 3, 6, 12, 18 and 24 months	Clinical and Radiographic	iRoot BP Plus, Calcium hydroxide	Traumatic	Immature permanent	Reversible pulpitis
Yildirim C et al., 2016 [53]	Turkey	NRS	140	24 months	Clinical and radiographic	Formocresol, MTA, Portland cement, enamel matrix derivative	Carious	Primary	Reversible pulpitis

Descriptive statistics were used to characterize the included papers, and narrative synthesis was undertaken to explain the results. Categorical data were summarized in frequencies and percentages, and numerical data in means and SD. Statistical analysis was performed using SPSS V.25.0 for Mac (IBM).

## Results

**Fig. 1:**
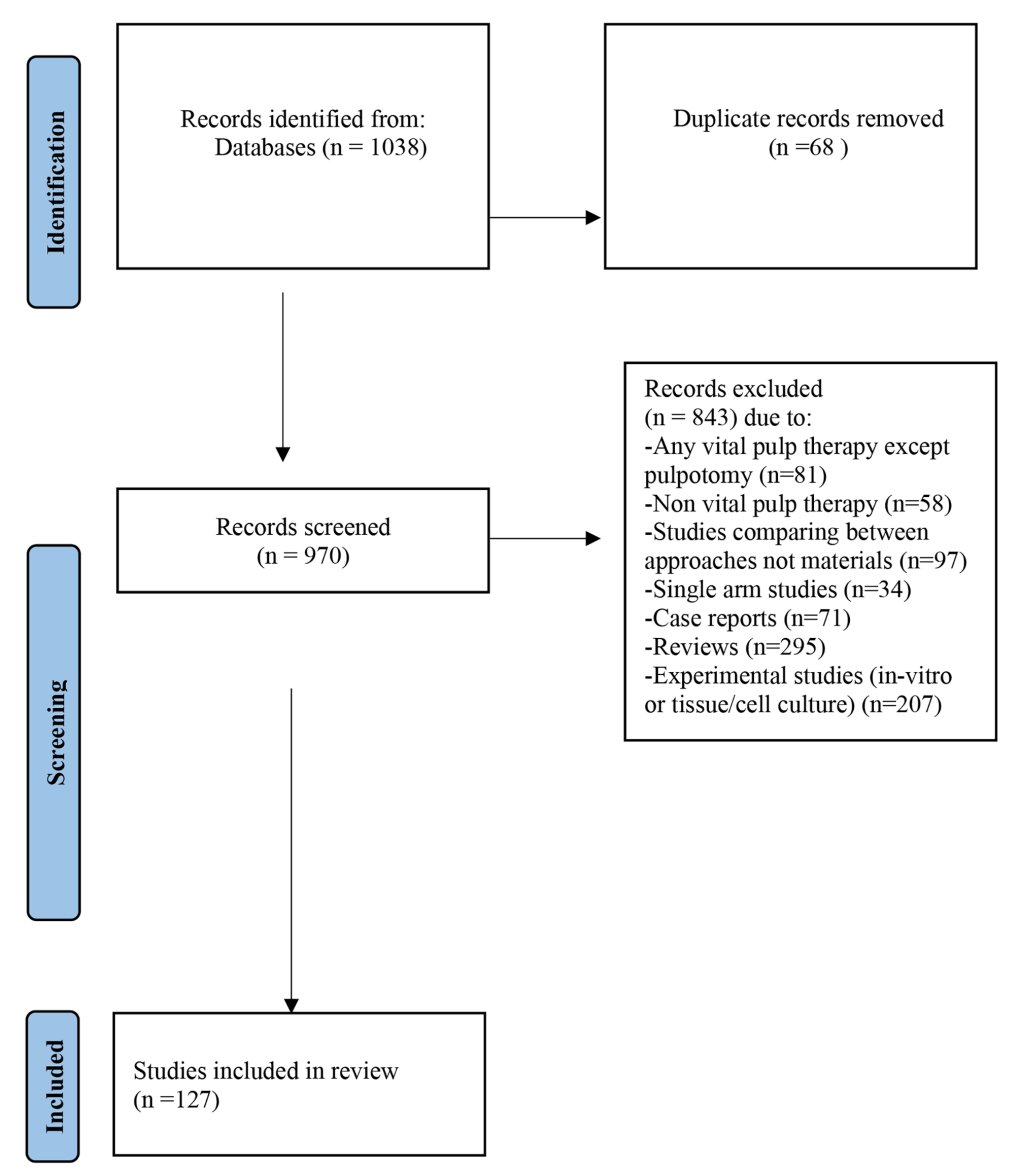
Flow chart of the reviewing process

The search identified 1038 potentially relevant records from all databases included. After removing the duplicates, 982 articles were screened by two independent reviewers to assess eligibility, and any conflict was resolved by a third reviewer. A total of 127 studies fulfilled all the inclusion criteria and were included in the current scoping review while 843 articles were excluded due to lack of adherence to the inclusion criteria. The flow chart of the review process is shown in Fig. 1 [16].

### Study sample, design, and outcome assessment method

Table 3 presents the general characteristics of the included studies. The sample size ranged from 17 to 469 participants with mean $\pm$SD: 84.$9\pm$71.6. Most of the studies (84.3%) were randomized controlled trials. The highest percentage of the studies had follow-up duration for 12 months (48%) followed by those who had follow-up duration more than 1 year (37.8%). The outcome measured in most of the studies (90.6%) were clinical and radiographic, few studies (5.5%) measured both clinical and histological outcomes. Majority of the studies (67.7%) involved primary teeth as compared to 32.3% for the permanent teeth. Among those, 22.8% of the studies used mature teeth and only 9.4% of the studies used immature teeth. Carious exposure was the most common type of pulp exposure accounting for 89.8% of the studies. Regarding the pre-operative pulpal status, the majority of the teeth in the screened studies had a diagnosis of reversible pulpitis (81.9%) while a small number were diagnosed with irreversible pulpitis (10.2%).

### Interventions

**Fig. 2:**
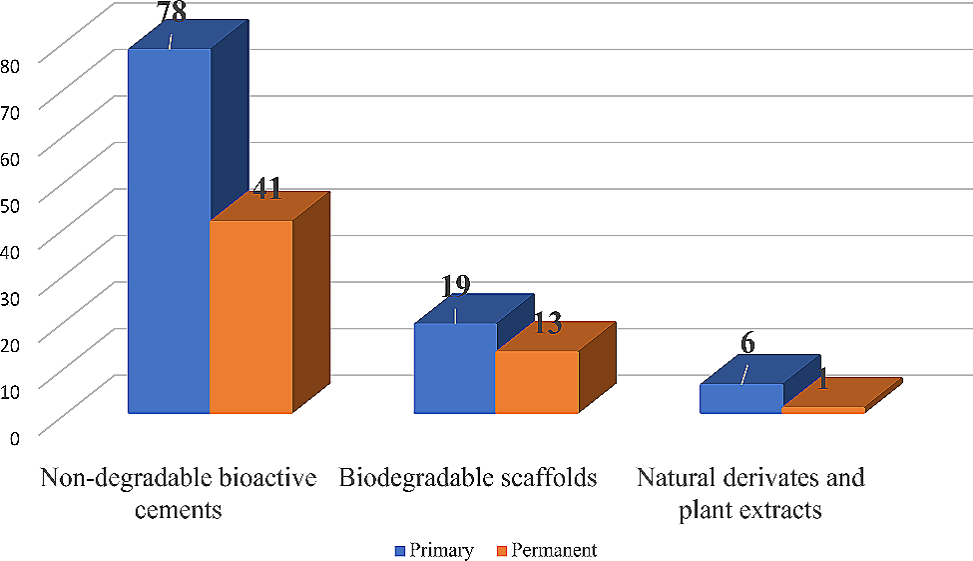
Distribution of studies among different groups of bioactive pulpotomy agents

Figure 2 presents the number of studies for different groups of bioactive pulpotomy agents of both primary and permanent teeth, and it shows that 78 studies used non-degradable bioactive cements in primary teeth as compared to 41 in permanent teeth, while biodegradable scaffolds were used by 19 studies involving primary teeth and 13 studies in permanent teeth, natural derivates and plant extracts studies presented six studies in primary teeth and only one study in permanent teeth.

**Fig. 3:**
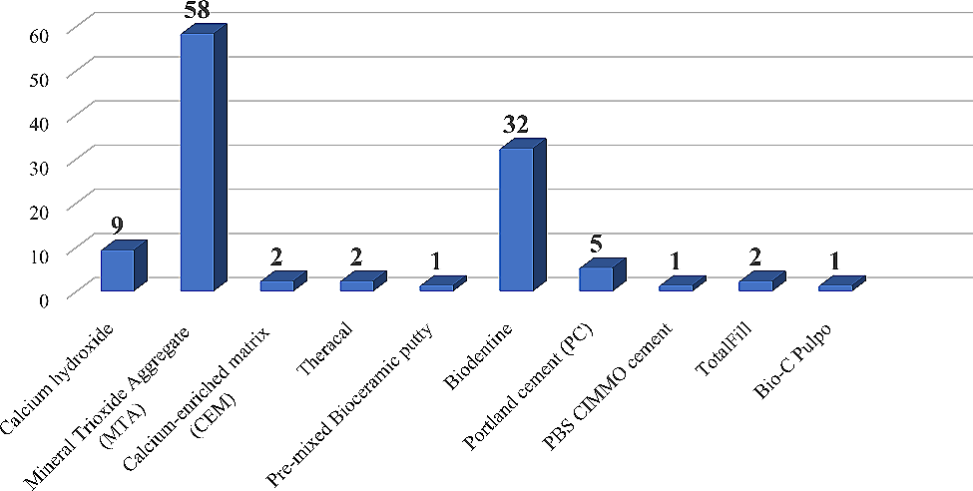
Number of studies of different non-degradable bioactive cements in primary teeth

Among the studies that used non-degradable bioactive cements in primary teeth, the majority (58 studies) used MTA, followed by biodentine (32 studies) and calcium hydroxide (9 studies). Other materials like Portland cement, calcium-enriched mixture cement (CEM), Theracal, Totalfill, pre-mixed Bio Ceramic putty, PBS CIMMO cement and bio-c pulpo were used less frequently, as shown in Fig. 3.

**Fig. 4:**
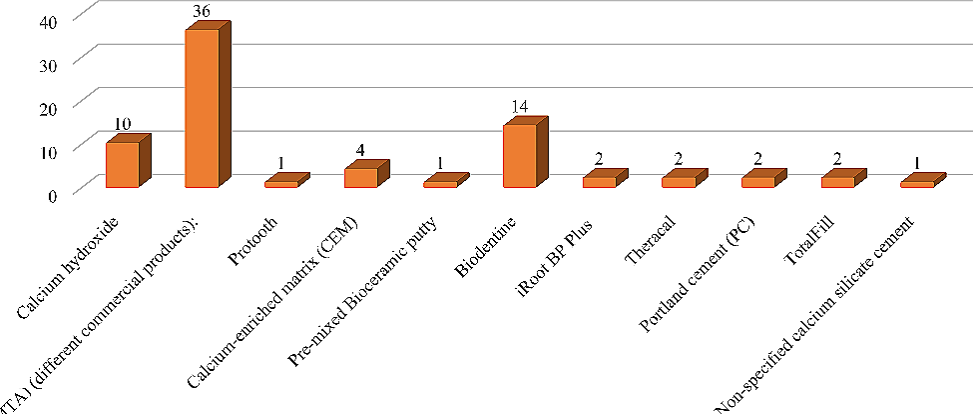
Number of studies of different non-degradable bioactive cements in permanent teeth

Figure 4 presents the number of publications included which used non-degradable bioactive cements in permanent teeth. It shows that the majority (36 studies) used MTA, followed by biodentine (14 studies) and calcium hydroxide (10 studies). Other materials like calcium-enriched mixture cement (CEM), Totalfill, Portland cement, TheraCal, iRoot BP plus, pre-mixed Bio ceramic putty, Protooth and non-specified calcium silicate cement were used less frequently.

**Fig. 5:**
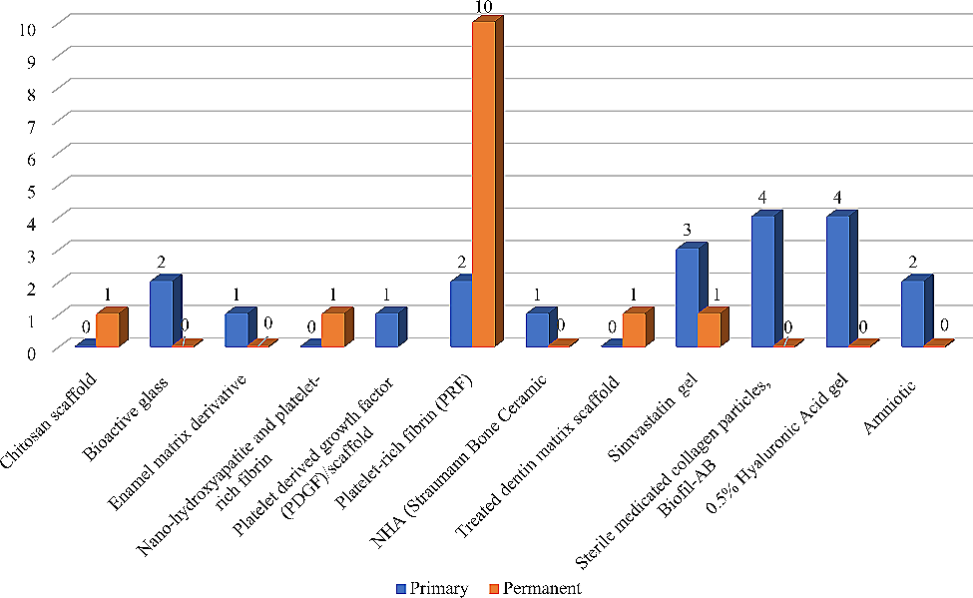
Number of studies of different biodegradable scaffolds in primary and permanent teeth

Among the included studies which used biodegradable scaffolds in primary teeth, the most common materials used were 0.5% Hyaluronic acid gel (4 studies), Biofill-AB (4 studies), Simvastatin gel (3 studies), platelet-rich fibrin (2 studies), Bioactive glass (2 studies) and Amniotic (2 studies). While in studies involving permanent teeth, platelet-rich fibrin (PRF) was the most common material used (10 studies), other materials like chitosan scaffold, nano-hydroxyapatite, treated dentin matrix scaffold and Simvastatin gel were used with less frequency (Fig. 5). The coronal sealing materials used in direct contact with these biodegradable scaffolds included glass ionomer cement (8 studies) and zinc oxide eugenol (12 studies) mainly for primary teeth. On the other hand, calcium hydroxide (1 study), Portland cement (1study), MTA (6 studies), and biodentine (3 studies) were mainly used in permanent teeth. In 5 of the studies, the coronal sealing material was not mentioned. Additionally, some studies used different sealing materials for different arms of the same study (Table 4).

**Table 3:** Characteristics and outcomes of the included studies

		**Mean (SD)**
Sample size		84.9 (71.6)
		**n (%)**
Study design		
	Randomized controlled trial	107 (84.3%)
	Non-randomized trial	20 (15.7%)
Follow-up duration		
	Less than 6 months	5 (3.9%)
	6 months	11 (8.7%)
	9 months	2 (1.6%)
	12 months	61 (48%)
	More than 1 year	48 (37.8%)
Outcome measured		
	Clinical and radiographic	115 (90.6%)
	Clinical, radiographic and histological	7 (5.5%)
	Histological	2 (1.6%)
	Clinical, radiographic and inflammatory	1 (0.8%)
	Histological and immunohistochemistry	1 (0.8%)
	Clinical, radiographic and microbiological	1 (0.8%)
Primary/permanent teeth		
	Primary teeth	86 (67.7%)
	Permanent teeth	
	Mature teeth	29 (22.8%)
	Immature teeth	12 (9.4%)
Pulp exposure type		
	Carious	114 (89.8%)
	Traumatic	6 (4.7%)
	Carious or traumatic	4 (3.1%)
	Sound tooth indicated for extraction	3 (2.4%)
Pre-operative pulp status		
	Normal pulp	5 (3.9%)
	Reversible pulpitis	104 (81.9%)
	Irreversible pulpitis	13 (10.2%)
	Reversible or Irreversible pulpitis	3 (2.36%)
	NOT MENTIONED	2 (1.57%)

**Table 4:** Coronal sealing materials used in studies employing biodegradable scaffolds

	Author, Year	Type of Exposure	Type of teeth used	Materials used	Coronal Sealing Materials
1	Abd Al Gawad R and Hanafy R. 2021 [55]	Carious OR traumatic	Primary	NHA (Straumann Bone Ceramic), MTA, Formocresol	stainless crown cemented with glass ionomer
2	Abdel Maksoud E, 2023 [56]	Carious	Primary	Hyaluronic Acid, Amniotic Membrane Allograft, Mineral Trioxide Aggregate	zinc oxide eugenol
3	Aboul Kheir M et al., 2020 [57]	Carious	Permanent	Chitosan scaffold, MTA	MTA
4	Aljabban et al., 2021 [133]	Sound premolar teeth scheduled for orthodontic extraction	Permanent	MTA, PRF	MTA
5	Anandan V et al., 2021 [70]	Carious	Primary	Formocresol, BioFil-AB Collagen Particles	zinc oxide eugenol and glass ionomer cement
6	Aripirala M et al., 2021 [71]	Carious	Primary	Simvastatin gel, 940 nm diode laser	resin-modified glass ionomer cement
7	Awad S, 2021 [74]	Carious	Infected immature permanent molars	Biodentine, Calcium Hydroxide, PRF	NOT MENTIONED
8	Bayoumi N, 2022 [77]	Carious	Primary	Sterile medicated collagen particles, Biofil-AB, Biodentine	NOT MENTIONED
9	Chak R et al., 2022 [84]	Carious	Primary	3Mixtatin, MTA	glass ionomer cement and stainless crowns
10	Eid A et al., 2022 [90]	Carious	Immature permanent molars	MTA (MM-MTA), nano-hydroxyapatite, platelet-rich fibrin	IRM for nano-hydroxyapatite and zinc oxide eugenol for the PRF group
11	Elhamouly Y et al., 2021 [18]	Carious	Primary	Biodentine, bioactive glass	glass ionomer
12	Elheeny A, 2023 [94]	Carious	Immature permanent teeth	Simvastatin, MTA	NOT MENTIONED
13	Elsayed S, 2023 [136]	Carious	Primary	Biofil-AB, Biodentine	glass ionomer
14	Eltantawy W, 2023 [97]	Carious	Primary	Biodentine, hyaluronic acid, Formocresol	zinc oxide eugenol
15	Haideri S et al., 2021 [108]	Carious	Primary	Formocresol, Mineral Trioxide Aggregate, Electrocautery, Bioactive Glass	IRM and stainless-steel crown
16	Ildes G et al., 2022 [110]	Carious	Primary	0.5% Hyaluronic Acid gel, Formocresol, 20% Ferric sulphate	zinc oxide eugenol and composite/stainless steel crown
17	Kakarla P et al., 2013 [102]	Sound and indicated for extraction	Primary	Pulpotec, Biofil-AB	zinc oxide eugenol and glass ionomer cement
18	Keswani D et al., 2014 [122]	Carious	Immature Permanent	MTA, PRF	zinc oxide eugenol and amalgam
19	Kumar V et al., 2016 [124]	Carious	Permanent	Calcium hydroxide, MTA, platelet-rich fibrin	MTA
20	Manhas M et al., 2019 [135]	Carious	Primary	MTA, Calcium hydroxide, PRF	either calcium hydroxide or MTA
21	Mehrvarzfar P et al., 2017 [134]	Traumatic	Permanent third molars	MTA, Treated dentin matrix scaffold	resin-modified glass ionomer cement
22	Mentes A, 2020 [22]	Carious	Primary	Formocresol, ferric sulphate, and 0.5% hyaluronic acid	zinc oxide eugenol, composite and stainless-steel crown
23	Nageh M, 2021 [23]	Carious	Permanent	Biodentine, PRF, MTA, Portland cement	PRF covered with portland cement or MTA or biodentine
24	Najmi N, 2022 [25]	Carious	Permanent	PRF, MTA, calcium hydroxide	NOT MENTIONED
25	Patidar S et al., 2017 [138]	Carious	Primary	PRF, MTA	zinc oxide eugenol and glass ionomer cement, stainless steel crown
26	Prasad M et al., 2017 139]	Carious	Primary	Amniotic, Formocresol	zinc oxide eugenol
27	Sherif R, 2019 [131]	Carious	Permanent	PRF, Biodentine, diode laser	biodentine, glass ionomer and composuite
28	Singh R et al., 2020 [38]	Carious	Permanent	Calcium hydroxide, MTA, PRF	NOT MENTIONED
29	Suez Canal University, 2022 [40]	Carious	Primary	Biodentine, Simvastatin	glass ionomer cement and stainless-steel crown
30	Surinder et al.,2021 [132]	Carious	Permanent	MTA, Biodentine, Platelet Rich Fibrin	PRF/MTA, PRF/Biodentine
31	Venugopal N et al., 2019 [137]	Carious	Primary	Formocresol, propolis, Platelet derived growth factor (PDGF)/scaffold	collagen membrane then glass ionomer and stainless-steel crown
32	Yildirim C et al., 2016 [53]	Carious	Primary	Formocresol, MTA, Portland cement, enamel matrix derivative	zinc oxide eugenol and glass ionomer cement

**Fig. 6:**
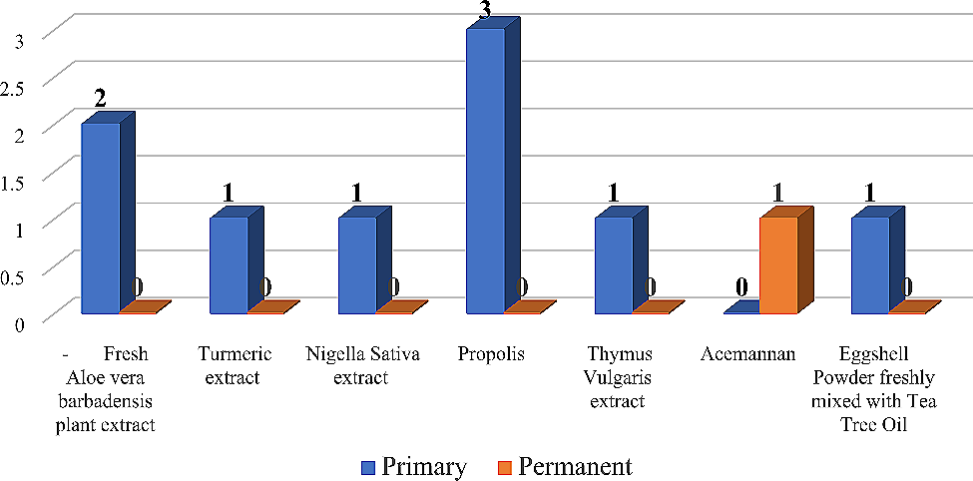
Number of natural derivates and plant extracts studies in primary and permanent teeth

Natural derivates and plant extracts presented the least number of studies. Concerning primary teeth, three studies used *propolis*, two studies used fresh *aloe vera barbadensis* plant extract and only one study was found for each of these materials: *turmeric extract*, *nigella sativa* extract, *thymus vulgaris* extract and *egg-shell powder mixed* with *tea tree oil*. As for permanent teeth, only one study was reported, and it used *acemannan* (Fig. 6).

### Time distribution of the included studies

**Fig. 7:**
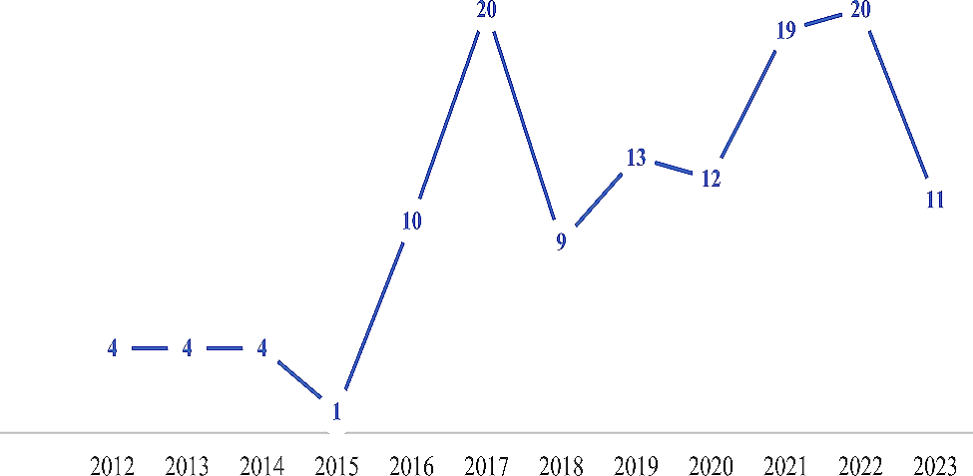
Trend in the number of publications using bioactive cements and biodegradable scaffolds from 2012 to 2023

The number of articles published increased generally between 2012 (4 studies) and 2023 (11 studies), indicating a growing interest in and expansion of the research field of bioactive pulpotomy agents. The peak of the studies was in 2017 and 2022, accounting for 20 studies (Fig. 7).

### Global distribution of the included studies

**Fig. 8:**
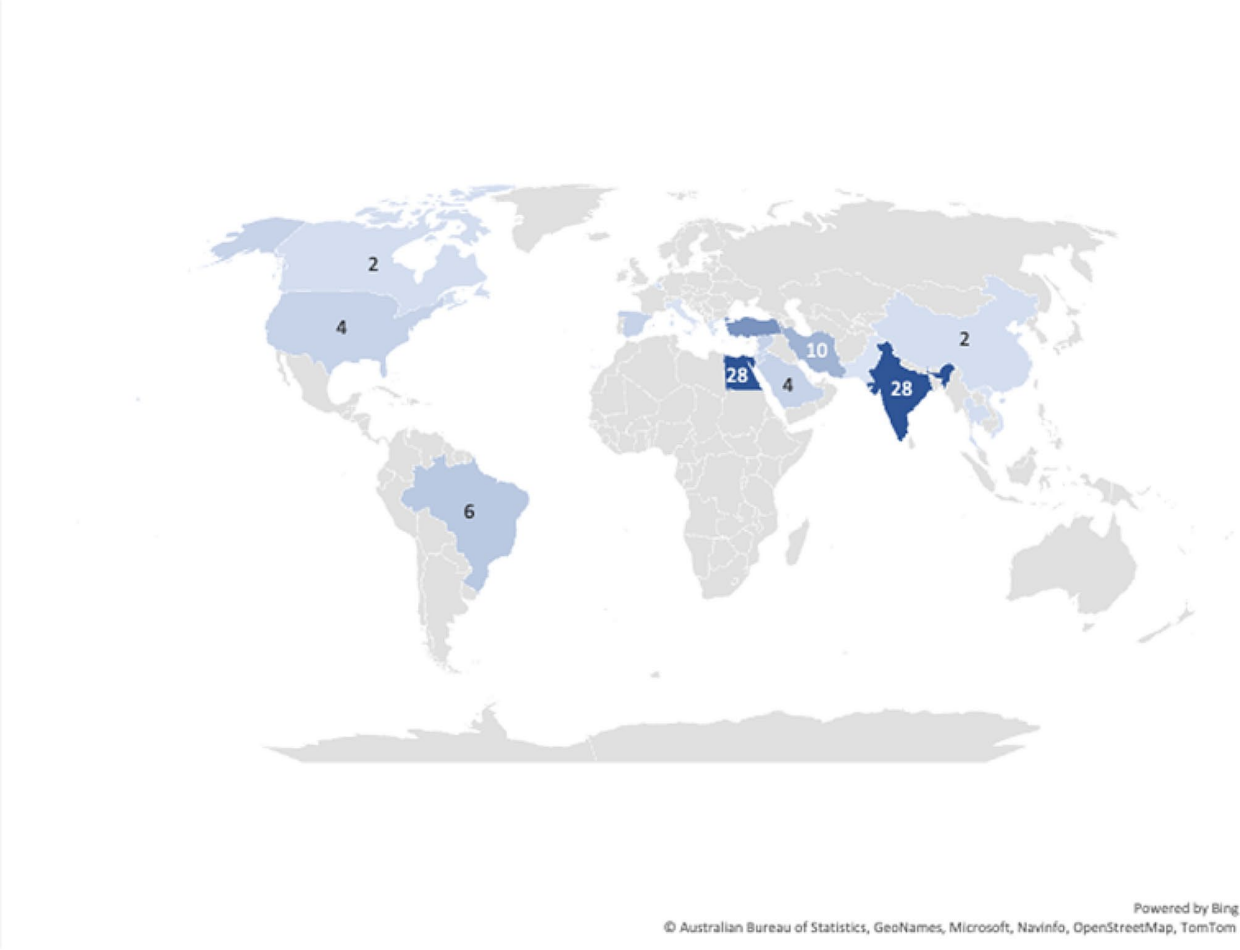
Map of the studies included in the scoping review

Concerning publishing countries; India, Egypt, Turkey, and Iran were found to have the highest total number of published articles (28, 28, 16 and 10 studies, respectively). Other studies were conducted in smaller numbers in Brazil (6 studies), United States (4 studies), Syria (4 studies), Spain (4 studies), Israel (4 studies), Korea (4 studies), Kingdom of Saudi Arabia (4 studies), Jordan (3 studies), Thailand (2 studies), Belgium (2 studies), Canada (2 studies), China (2 studies), Italy (1 study), Pakistan (1 study), Greece (1 study) and Vietnam (1 study) (Fig. 8). Nineteen articles from the total number included in the review are registered clinical trials that are still in the recruitment phase; fourteen of them are being conducted in Egypt, two in India, one in Spain, one in Jordan and one in Pakistan.

## Discussion

Over the past decade, there has been a paradigm shift in the realization that an inflamed pulp may be worth saving. Advancements in the fields of tissue engineering and biomaterials have made preservation and regeneration of the dentin-pulp complex the most sought-after goals of vital pulp therapy strategies. Although the evolution of biomaterials since the discovery of calcium hydroxide has been immense and revolutionary, the unique spatiotemporal nature of the dentin-pulp complex poses multiple challenges. This is further complicated by the inherent anatomical, physiological, and biological differences between the primary and the permanent dental pulps [17]. Furthermore, while the required outcome may be the same, indications and outcome assessment methods for pulpotomy procedures in primary and permanent teeth may be quite different. Indeed, in an era where personalized patient care will represent the future of medicine, bioactive vital pulp therapy agents that aim to regenerate anatomical and functional tissues like the native tissue are continuously being developed. These agents and strategies must therefore be carefully tailored not only to whether the tooth is primary or permanent but also according to the developmental and inflammatory status of the tooth in question [18].

Although pulpotomy procedures for primary teeth have long been practiced, the concept of a pulpotomy for a mature permanent tooth has only recently been addressed. Hence, we aimed to focus more on the last 10 years in which a peak in the publication of these papers was noted. Additionally, the use of bioactive cements and biodegradable scaffolds in randomized clinical trials focusing on pulpotomy is relatively new. Therefore, the goal of this scoping review was to elucidate the present knowledge gap and highlight the need for clear decision-making guidelines regarding outcome assessment methods of pulpotomy procedures utilizing regenerative agents in primary and permanent teeth. It was designed and reported with reference to the recently updated JBI scoping review guidelines [15, 19] and Preferred Reporting Items for Systematic Reviews and Meta-Analyses Scoping Review extension (PRISMA-ScR) [2, 20], and was reinforced by the diverse expertise of the authors who include methodologists, analysts, and clinicians sharing an intrigue in evidence-based health care.

While planning this review, the language was restricted to English only to avoid potential confusion in interpretation of data during translation of full text articles. As for exclusion of studies comparing different vital pulp therapy techniques, we wanted to focus the attention of this review on different materials without having the confounding variables of different procedural parameters. Furthermore, we intended to target randomized and non-randomized controlled clinical trials only to provide an overview of the available highest level of clinical evidence to answer the research question, and to determine where further research may be indispensable in this field. We did not set limits for the follow-up periods to include short- and long-term clinical, radiographic, as well as histological and inflammatory assessments. Most of the screened clinical trials (85.8%) comprised 12 months and longer follow-up intervals [18, 21–127]. As the objective of this review was focused on outcome assessment rather than treatment success, which is highly dependent on the initial inflammatory pulp status, we did not restrict our search according to the type of exposure being carious or traumatic to retrieve as many trials as possible in our search in primary and permanent teeth. Additionally, included studies did not stratify the outcomes according to the type of exposure. Remarkably, the pre-operative pulpal status was mainly distinguished as “reversible pulpitis” for both primary and permanent teeth [18, 21, 22, 26, 28–36, 39, 40, 43, 44, 47–56, 58–60, 62–71, 75–123, 125–127, 135–139, 150, 151, 155–157, 167, 168]. On the other hand, teeth categorized with “irreversibly inflamed pulps” were indicated for pulpotomy only for mature permanent teeth [23, 25, 27, 37, 38, 45, 46, 57, 61, 72, 124, 131, 132]. The small percentage of studies performed in mature permanent teeth with irreversible pulpitis highlights this new trend in treatment of the inflamed pulp.

With regards to the total number of studies, the fact that the studies in primary teeth represented almost double those in permanent teeth again clearly reflects that the pulpotomy trend for mature permanent teeth is a new direction in treatment. This is owing to the fact that the pulpotomy procedure is the preferred treatment for preserving the vitality of an asymptomatic cariously exposed primary or immature permanent tooth as dictated by the American Academy of Pediatric Dentistry [128] and is a newly prospective substitute for root canal treatment in managing mature or immature permanent teeth with carious pulp exposures, even with irreversible pulpitis [129]. This was also augmented by the recent position statement from the European Society of Endodontology [130] who recommended minimally invasive vital pulp therapy (VPT) for permanent teeth.

Interestingly, while studies on both primary and permanent teeth displayed a high tendency to use bioactive agents, more than 31.7% (13/41) of studies on permanent teeth implemented biodegradable scaffolds [23, 25, 38, 57, 74, 90, 94, 122, 131–134]versus only 22.1% (19/86) of the studies conducted on primary teeth [18, 22, 40, 53, 55, 56, 70, 71, 77, 84, 97, 102, 108, 110, 135–139]. This could reflect the recent nature of the use of pulpotomy procedures as a permanent treatment modality in mature permanent teeth, which coincides with the recent boom in the development and optimization of a wide variety of bioactive agents [3, 5, 6]. This could also be attributed to the higher need for retaining the permanent teeth throughout life of the patients. Furthermore, it might highlight the differences in outcome assessment methods and follow up duration required following pulpotomy in primary or permanent teeth.

For primary teeth, the main objective of pulpotomy procedures is to keep the tooth symptom-free until the successor tooth erupts [7]. Hence, it is seldom required to aim to regenerate the damaged tissue but rather sustain the condition of the vital pulp until the time of shedding. Indeed, many studies in primary teeth do not consider minor radiographic changes as a reason for further intervention since the tooth can function and the patient has no signs or symptoms [140–143]. However, this concept fails to consider the duration of time that this failure may require. Additionally, it has been shown that the inflammatory milieu within the pulp may be influenced by the active conditions of physiologic tooth resorption, and the contrary may also be true [144–147]. Root resorption is one of the most frequently reported reasons of failure in primary teeth which may again highlight a continued inflammatory trigger even following the pulpotomy procedure [33, 99, 148]. Aiming to regenerate the lost dentin-pulp tissue and restore nociception and immune defense within the pulp may create an inflammation-free environment, allowing the natural process of shedding and eruption. On the other hand, the goal of partial or complete pulpotomy procedures in permanent teeth is to remove the coronally inflamed or infected pulp and preserve the remaining normal or reversibly inflamed radicular pulp. It also aims to promote healing and repair of the remaining vital tissue, not as a temporary treatment but rather as a long-term predictable treatment like conventional root canal treatment [130, 149].

The recent rise in the implementation of biodegradable scaffolds for pulpotomy procedures demonstrates the rapid transition in knowledge and understanding of the dentin-pulp complex from preservation to regeneration. There are also a handful of studies that have used natural derivatives and plant extracts indicating a tendency towards using readily available, naturally healing materials that not only have therapeutic potential but are also cost-effective and environment friendly [35, 50, 93, 116, 125, 137, 150]. Indeed, the use of these extracts in primary teeth has long preceded their use as palliative and healing agents in the permanent dentition.

The evolution of bioactive cements is clearly demonstrated by the results of this study in that most clinical studies utilized either MTA or more recently biodentine [18, 21, 23–30, 32, 33, 35–50, 53–66, 68, 69, 72–101, 103–109, 111–127, 131–136, 138, 151–157]. MTA has been advocated as the new gold standard for pulpotomy procedures. Certainly, almost all of the included studies used MTA for one of the control arms [21, 23–30, 32, 33, 35, 37–39, 41–46, 48–50, 53–70, 72, 73, 75, 76, 78, 79, 81–85, 87–90, 92–96, 98–104, 106–109, 111–127, 132–136, 138, 151–154, 156, 157]. It has excellent potential as a pulpotomy medicament, as it is highly biocompatible, with regenerative potential and effective induction of dentinal bridge formation. Furthermore, a recent study suggested MTA to be a useful material in both infected and uninfected pulp tissue [158] with low toxicity [159] and no adverse effects on permanent successors [160]. The years of clinical experience have revealed some disadvantages of MTA that occur in practice, such as long setting time, potential of discoloration and lengthy procedure. Biodentine seems to have superior properties in that it is more biocompatible, has better handling properties, produces more predictable dentin bridges, and shows comparable treatment outcomes to MTA [59, 82, 98, 161].

Out of the 13 studies that used biodegradable scaffolds in permanent teeth, the majority were conducted using PRF [23, 25, 38, 74, 122, 124, 131–133, 138]. On the other hand, the 19 studies conducted on primary teeth utilized a variety of scaffolds with 0.5% hyaluronic acid gel being the most implemented [22, 56, 97, 110]. This could be due to the rising concept of using regenerative agents for pulpotomy procedures in primary teeth. The use of PRF as a scaffold for permanent tooth pulpotomy stems from the rise in the use of PRF and other platelet-derived concentrates as scaffolds for regenerative endodontics [162, 163]. Platelet rich fibrin is a second-generation platelet-rich concentrate that relies on the body’s own clotting mechanisms without the addition of extrinsic factors to trigger coagulation. The clinical protocol for producing autologous PRF is relatively simple, cost-effective, and reproducible. In comparison to platelet-rich plasma (PRP) and other platelet-derived concentrates, PRF can provide a more sustained release of growth factors which can aid in stem cell recruitment, angiogenesis, and cell proliferation and differentiation [164, 165]. One drawback which may limit the use of PRF and other concentrates for primary teeth pulpotomy is that its procurement may be considered an invasive procedure from children often requiring withdrawing 5–10 cc of blood [165, 166].

A notable observation in this study was that most pulpotomized primary teeth as well as young permanent teeth, capped with biodegradable scaffolds in the screened studies, were covered with zinc oxide eugenol and/or glass ionomer cement followed by stainless steel crowns. This could be because zinc oxide eugenol is regarded as a preservative material not capable of initiating a reparative process in addition to being the material of choice for standard of care procedures in pulpotomized primary teeth [128]. On the other hand, studies in mature permanent teeth that used biodegradable scaffolds/agents mainly utilized mineral trioxide aggregate, biodentine or other calcium silicate cements for sealing. These materials have also been recommended by recent guidelines as vital pulp capping materials [130]. Undoubtedly, the choice of sealing material may have a profound negative effect on the outcome of healing, further advancing the inflammatory process and eventually contributing to failure of the procedure [18].

Although this review clearly shows that numerous well-conducted clinical studies have evaluated pulpotomy outcomes with bioactive agents both in primary and permanent teeth, more than 90% of the screened trials [18, 21–101, 103–115, 125–127, 131–139, 150–153, 155, 156, 167, 168] assessed the pulpotomy treatment outcome via subjective clinical and radiographic parameters.However, recent data has shown that the initial inflammatory status of the pulp is perhaps the only true determining factor that affects the outcome of treatment. Around only 10% of the studies mapped in this review performed histological analysis or attempted to measure inflammatory biomarkers [18, 28, 56, 87, 102, 109, 133, 134, 137, 152, 154, 157]. While histological analysis is of course not possible and, in fact, unwarranted in most clinical trials, it remains the only measure of the actual condition of the pulp [169].

Several recent studies have shown that dentinal fluid and pulpal blood of teeth with inflamed pulps may contain elevated levels of pro-inflammatory markers that can determine the inflammatory status of the pulp [170, 171]. Whilst numerous efforts have been made recently to link biological markers of inflammation (quantitative measure of inflammatory cytokines) to the status of pulp, scarce evidence was identified among the screened trials in this regard. Only one published study [18] and one completed registered clinical trial [87] assessed the relationship between markers of pulp inflammation and the outcome of pulpotomy treatment. Therefore, this review highlighted the gap in the literature with respect to inflammatory assessment of the preoperative pulpal status and its correlation with the pulpotomy outcomes. This has triggered the search for specific pulpal markers and the development of chair-side detection kits that may better help in assessing the eligibility of teeth for pulpotomy procedures and thereby provide the basis for better diagnosis and predictable treatment outcomes.

Another important fact to take into consideration is the duration of follow-up. Pulpotomy procedures have been long practiced in primary teeth, thus providing long term data. However, there are very few studies in permanent teeth with more than 2-4-year follow-up, which is considered moderate-term follow-up at best. Most of the studies were content to hit the 12-month recall [18, 22–25, 27, 32, 37–44, 47, 50, 51, 54–57, 61, 62, 64, 66–72, 77, 79, 80, 84, 85, 88, 90–93, 95, 96, 98, 100, 103–105, 108, 110, 112, 113, 116–118, 120, 124, 125, 127, 131, 132, 151]. This, however, does not allow ample time to assess important parameters such as incidence of root resorption, pulp canal obliteration, tooth survival or impact on quality of life [10, 11].

Two intriguing findings from the current scoping review are the trend of publications from 2012 to 2023 as well as the global distribution of studies. The increased implementation of vital pulp treatment strategies in the last ten years has started as a result of a global effort to diagnose accurately the pulp status of permanent teeth, to preserve pulp vitality, and to increase pulp survival. Regarding the number of publications, there appear to be two peaks: in 2017 and in 2022. The first peak appears to coincide with the initial surge of clinical trials published deeming pulpotomy as a permanent treatment modality for mature permanent teeth with symptomatic irreversible pulpitis, as evidenced by several studies published in that period (20/127) [24, 29, 30, 33, 42, 64, 79, 85, 109, 114–116, 118, 120, 134, 138, 139, 152, 154, 167]. The second peak seems to correspond to the immediate post-pandemic phase. During the COVID-19 outbreak, clinical researchers were impaired by reduced access to health care, and clinical trials were suspended and postponed. Scientific research was then resumed as the world was vaccinated and access to health care was restored [172].

At the beginning of the pandemic and later throughout the rest of 2020 to 2021, most clinical recommendations for the emergency treatment of “hot” teeth or teeth with symptomatic irreversible pulpitis were to employ pulpotomy procedures when possible as a permanent treatment [173, 174]. The reduced invasive nature of the procedure and the ability to perform a pulpotomy in one visit, in addition to the reduced operative time, reduced the risks of infection with COVID-19 due to dental exposure. This, coupled with the apparent reduced cost of pulpotomies, seemed to help in convincing more practitioners to attempt this treatment, although it is still new and lacking long-term evidence. This behavior corresponds perfectly with the sharp peak in the number of publications and clinical trials reported in 2022 (20/127) [21, 25, 35, 37, 41, 48, 61, 63, 73, 76, 77, 84, 90, 96, 98, 110, 112, 127, 156]. The other remarkable finding that coincides with the publication trends is the global distribution, where most studies are distributed in low-to-middle-income countries in the middle east and Asia, particularly in Egypt and India. This global distribution again reflects how adopting pulpotomy procedures as permanent treatments, especially in mature permanent teeth, can present multiple benefits especially where resources are limited.

A significant strength of this scoping review is the demonstration of compliance with the recently updated JBI scoping review guidelines [15, 19] and PRISMA-ScR [2, 20]. Moreover, to our knowledge, this is the first scoping review to address the outcome assessment methods of pulpotomy procedures in both primary and permanent teeth using regenerative non-degradable bioactive cements and biodegradable tissue engineering scaffolds. Another point of strength in this review is that it included grey literature, especially registered clinical trials in the National Institute of Health (NIH) database. The inclusion of grey literature allows a more objective perspective on the status of the evolution of new concepts in treatment.

On the other hand, some limitations related to the methodology were encountered while conducting this review. Only primary research (controlled trials, randomized controlled trials) was included while uncontrolled trials, case reports, case series, systematic reviews, position statements, and clinical guidelines were not. Despite our search being guided by an expert librarian, our electronic literature search was bounded to MEDLINE (via Pubmed), Web of Science, Scopus, Proquest, and clinicaltrials.gov in an attempt to limit the number of articles being scanned; however, this might have led to missing some evidence that address the review questions and objectives present in other search engines.

Our review also revealed significant knowledge gaps, including a scarcity of studies conducted on permanent teeth and a dearth of studies establishing a correlation between actual inflammatory status of the pulp and treatment outcomes. Therefore, long term evidence from well-conducted clinical trials is still needed, as well as the development of a set of predictable outcome measures and the interpretation of outcomes in terms of both treatment success and tooth survival. It is also crucial when designing new biodegradable scaffolds, for promoting tissue regeneration following pulpotomy procedures, to tailor their properties according to the inflammatory milieu and whether they will be designed for usage in primary or permanent teeth.

## Conclusions

Within the limitations of this scoping review, the findings underscored that evaluation methods of pulpotomy procedures using regenerative agents in primary and permanent teeth, over the past decade, primarily focused subjectively on clinical and radiographic outcomes. On the other hand, there are few studies that objectively assessed the pulpal inflammatory status. Among various materials, MTA emerged as the most frequently utilized capping material followed by biodentine. However, a limited number of studies incorporating biodegradable scaffolds for pulpotomy procedures were found. Furthermore, the results indicated a recent surge in publications originating in low-to-middle-income countries; hence, indicating a widespread implementation potential for pulpotomy procedures in both dentitions.

### Electronic supplementary material

Below is the link to the electronic supplementary material.

Supplementary Material 1

Supplementary Material 2

## Data Availability

No datasets were generated or analysed during the current study.

## Abbreviations

OHRQoL: Oral health-related quality of life
MTA: Mineral trioxide aggregate
JBI: Joanna Briggs Institute
CEM: Calcium-enriched mixture
PRF: Platelet-rich fibrin
PRISMA-ScR: Preferred Reporting Items for Systematic Reviews and Meta-Analyses Scoping Review extension
VPT: Vital pulp therapy
PRP: Platelet-rich plasma
NIH: National Institutes of Health
RCT: Randomized clinical trial
NRS: Non-randomized study
