# Supplementary material for: Outcome assessment methods of bioactive and biodegradable materials as pulpotomy agents in primary and permanent teeth: a scoping review
Source: BMC Oral Health. 2024 Apr 27;24:496. doi: 10.1186/s12903-024-04221-w (PMC11055312; doi:10.1186/s12903-024-04221-w)
Supplement: Supplementary file 2 — Supplementary Material 2 [file 12903_2024_4221_MOESM2_ESM.docx]

**List of Materials Used in The Current Scoping Review**

| **Non-degradable bioactive cements** | **Biodegradable scaffolds** | **Natural derivatives and plant extracts** | **Others** |
| --- | --- | --- | --- |
| 1. Calcium hydroxide 2. Mineral Trioxide Aggregate (MTA) (different commercial products):  - MedCem MTA - MTA Angelus - MTA plus - MTA HP Repair - Fast-setting MTA ENDOCEM - NuSmile NeoMTA - OrthoMTA - ProRoot MTA - RetroMTA  1. Protooth 2. Calcium-enriched matrix (CEM) 3. Pre-mixed Bioceramic putty 4. Biodentine 5. iRoot BP Plus 6. Theracal 7. Portland cement (PC) 8. Non-specified Calcium silicate cements 9. PBS CIMMO cement 10. TotalFill 11. Bio-C Pulpo | - Chitosan scaffold - Bioactive glass - Enamel matrix derivative - Nano-hydroxyapatite and platelet-rich fibrin - Platelet derived growth factor (PDGF)/scaffold - platelet-rich fibrin (PRF) - NHA (Straumann Bone Ceramic - Treated dentin matrix scaffold with or without MTA - Simvastatin gel - Sterile medicated collagen particles, Biofil-AB - 0.5% Hyaluronic Acid gel - Amniotic membrane scaffold | - Fresh Aloe vera barbadensis plant extract - Turmeric extract - Nigella Sativa extract - Propolis - Thymus Vulgaris extract, - Acemannan - Eggshell powder mixed with tea tree oil | - Ferric sulfate - NaOCl - Pulpotec - Tempophore TM - Polyethylene glycol - Antioxidant mix |
